# State of the Art in 3D Culture Models Applied to Thyroid Cancer

**DOI:** 10.3390/medicina60040520

**Published:** 2024-03-22

**Authors:** Alessandro Prete, Antonio Matrone, Roberto Plebani

**Affiliations:** 1 Department of Clinical and Experimental Medicine, Endocrine Unit 2, University of Pisa, 56122 Pisa, Italy; alessandro.prete22@gmail.com; 2 Department of Medical, Oral and Biotechnological Sciences, “G. d’Annunzio” University, 66100 Chieti-Pescara, Italy; roberto.plebani@unich.it

**Keywords:** 3D culture models, organ-on-a-chip, thyroid-on-a-chip, gland-on-a-chip, thyroid cancer, microfluidic devices

## Abstract

Thyroid cancer (TC) is the prevalent endocrine tumor with a rising incidence, particularly in higher-income countries, leading to an increased interest in its management and treatment. While overall, survival rates for TC are usually favorable, advanced cases, especially with metastasis and specific histotypes, pose challenges with poorer outcomes, advocating the need of systemic treatments. Targeted therapies have shown efficacy in both preclinical models and clinical trials but face issues of resistance, since they usually induce partial and transient response. These resistance phenomena are currently only partially addressed by traditional preclinical models. This review explores the limitations of traditional preclinical models and emphasizes the potential of three-dimensional (3D) models, such as transwell assays, spheroids, organoids, and organ-on-chip technology in providing a more comprehensive understanding of TC pathogenesis and treatment responses. We reviewed their use in the TC field, highlighting how they can produce new interesting insights. Finally, the advent of organ-on-chip technology is currently revolutionizing preclinical research, offering dynamic, multi-cellular systems that replicate the complexity of human organs and cancer–host interactions.

## 1. Introduction

Thyroid cancer (TC) is the most common endocrine tumor, with nearly 44,000 new cancer diagnoses in 2022 [1]. Over the past four decades, its incidence has dramatically increased in higher-income countries, mainly due to overdiagnosis, but also driven by other factors such as obesity or the environment (e.g., endocrine disruptors) [2,3]. In particular, TC has been recognized as one of those cancer associated with excess adiposity by the International Agency for Research on Cancer (IARC), and an increase of 5 units in the body mass index has been associated with a 30% greater risk of TC [4,5]. Although this growing incidence is mainly due to low-risk TC [6], also advanced cases have increased over time, albeit at lower rates [6]. In particular, TCs with a larger diameter (>4 cm) have showed a significant increase, as well as cases with extrathyroidal extension and lymph-node metastases [7,8].

In the thyroid gland, neoplastic transformation occurs both in follicular and in parafollicular cells. Papillary (PTC), follicular (FTC), poorly differentiated (PDTC), and anaplastic TC (ATC) originate from follicular cells, while medullary TC (MTC) arises from parafollicular ones. Overall, thyroid malignancies have an excellent prognosis. According to Surveillance, Epidemiology, and End Results Program (SEER) data, the 5-year relative survival rate is 98.6% overall, 99.9% for localized, 98.3% for regional, and 54.9% for distant metastatic disease [9]. However, metastatic PTC and FTC, as well as specific histotypes—ATC, PDTC, and MTC—have worse prognosis. If global PTC 10-, 15-, and 20-year overall survival (OS) rates are 97, 95, and 90%, respectively [10], the presence of metastasis reduces survival, particularly in case of multi-organ and/or brain involvement and in old patients [10,11]. Prognosis of PDTC should be considered as intermediate between DTC and ATC with a five-year disease specific survival of 66%, according to retrospective or cohort studies [12]; distant metastasis control in PDTC is poor (59% at five years) and metastatic disease accounts for up to 85% of PDTC patients’ disease-related deaths [13]. At the end of this differentiation spectrum, ATC presents the worst OS of 0.79 years [14]; however, in case of presence of distant metastasis the 6-month cancer-specific survival is 19% [15]. Finally, MTC has a 10-year survival rate of 95.6%, 75.5% and 40%, in cases of localized, regional and metastatic disease, respectively [16]. Recently, *RET* mutations have been confirmed as negative prognostic factors in MTC patients, particularly indel mutations [17].

The management of advanced cases with worse prognosis is a challenge. The advent of systemic therapies in TC have an impact either in improving progression free survival (PFS) and also in prolonging OS, particularly in some histotypes (e.g., MTC and ATC [14,18]) and in some subgroups of patients with lower tumor burden [19] or better clinical conditions [20]. Furthermore, cases of exceptional response during systemic therapies have been reported both in ATC and MTC [21,22]. However, resistance occurring both at the beginning (primary resistance) and during (secondary resistance) systemic therapies is commonly reported [23]. Primary resistance has been observed with highly selective RET inhibitors in the presence of specific RET mutations (e.g., G810, Y806C/N and V738A) [24]. Secondary resistance could be fueled by activation of alternative pathways activation, acquisition of secondary mutations intrinsically resistance to targeted therapy or copy number amplification and the tumor microenvironment [23]. Traditional preclinical techniques such as two-dimensional (2D) cell cultures and mouse models have been widely used to address this unmet clinical need, with only partial success. New 3D culture models, such as transwell cultures, spheroids, organoids, and organ-on-a-chip technology, have emerged in recent years and are leading to significant progresses and continuous insights in oncological research. Here, we reviewed the use of preclinical models in TC research, highlighting their strengths and limitations and how these limitations could be overcome by using three-dimensional (3D) models.

## 2. Clinical Data Elicit the Need for New Preclinical Models of Thyroid Cancer

Targeted therapies induced a significant paradigm shift in treatment of advanced/metastatic TC. Currently, the drugs approved by the Food and Drug Administration and European Medicines Agency for the treatment of advanced/metastatic TC are sorafenib, vandetanib, cabozantinib, lenvatinib, selpercatinib and pralsetinib [25]. All these drugs were fully investigated in a classic preclinical model of TC. Sorafenib was shown to be able to downregulate MAPK pathway activation in thyroid cell lines harboring a BRAF mutation, inducing growth inhibition [26]. Vandetanib and cabozantinib were demonstrated to decrease cell growth via inhibiting RET phosphorylation and expression in RET-mutated PTC and MTC cell lines [27,28]. More recently, lenvatinib was shown to reduce cell proliferation of both PTC and MTC cell lines [29]. In 2018, selpercatinib and pralsetinib reduced cell proliferation in RET-mutated PTC and MTC cell lines [30,31]. Although the excellent results obtained in preclinical models, during treatment with these drugs, the tumor response was only partial and transient, due to resistance mechanisms.

In the phase 3, randomized, double-blind trial evaluating the efficacy and safety of sorafenib in radioactive iodine-refractory, locally advanced or metastatic differentiated TC (DECISION, clinicaltrialgov NCT00984282), the investigators observed a significant increase in progression free-survival (PFS) in the sorafenib arm compared with placebo (median 10.8 vs. 5.8 months, *p* < 0.001); however, the disease control rate—partial response plus stable disease—for ≥6 months was 54.1% [32]. Likewise, the phase 3, randomized, double-blind, trial evaluating efficacy and safety of lenvatinib in patients with iodine-131-refractory TC (SELECT, clinicaltrialgov NCT01321554), showed a significant increase in PFS compared with placebo arm (median 18.3 vs. 3.6 months, *p* < 0.001), although PFS at 12 and 24 months was 63.0 and 44.3%, respectively [33]. This resistance could be—at least in part—explained by the tumor microenvironment effect on cell cancer. Pericyte, a key element of the tumor microenvironment and crucial regulator of tumor micro-vascularity, has been shown to defend TC cells against targeted therapies [34]. In particular, they were observed to diminish the efficacy of vemurafenib (i.e., BRAF inhibitor) and sorafenib against PTC cell lines harboring the *BRAF* mutation via trombospondin-1/tumor growth factor 1 pathway (TSP1/TGFB1) [35]. Inhibition of the TSP1/TGFB1 pathway increases the sensitivity of cells to vemurafenib, sorafenib and combination therapy [35].

Both vandetanib and cabozantinib efficacy in advanced/metastatic MTC treatment was demonstrated in a randomized, placebo-controlled, double-blind, phase III study (ZETA clinicaltrialgov NCT00410761 and EXAM clinicaltrialgov NCT00704730) [36,37]: both induced a significant increase in PFS compared with the placebo arm (hazard ratio 0.46, *p* < 0.001, and 0.28, *p* < 0.001, respectively) [36,37]. However, in a real-world study, Koehler et al. showed that only 34% of patients treated with vandetanib had stable disease ≥ 24 weeks [38]. Moreover, Valerio et al. showed that early treatment with vandetanib, in the case of younger patients with a good ECOG performance status and symptomatic disease, seem to be the best predictors of a longer and durable response [39]. Likewise, in a randomized, double-blind noninferiority study to evaluate the efficacy of the cabozantinib 60 compared with 140 mg per day in patients with progressive, metastatic MTC, Capdevila et al. observed that 35% and 21% of patients treated with cabozantinib 60 mg/day and 140 mg/day discontinued the treatment because of progressive disease (EXAMINER trial, clinicaltrialgov NCT01896479 [40]). From a mechanistic point of view, Wang et al. showed that YES-Associated protein (YAP) plays a role in vandetanib resistance both in cell lines and mouse model of xenografted MTC [41]. The combination of vandetanib and YAP inhibitor induced a significant decrease in tumor volume compared with single treatment [41].

Selpercatinib and pralsetinib showed a significant response in *RET*-mutated TCs. In a phase 1–2 clinical trial involving adolescent and adult patients with TCs harboring an activating *RET* alteration (LIBRETTO 001, clinicaltrialgov NCT03157128), selpercatinib showed disease control (percentage of patients with complete response, partial response and stable disease) in more than 94% of patients with *RET*-mutant MTC (94% and 95% of patients already exposed to previous TKIs treatment and not, respectively) and 100% of patients with *RET*-mutated PTC at an independent and central review [42]. Moreover, more recently, Hadoux and Elisei et al. conducted a phase 3 randomized clinical trial comparing selpercatinib as first-line therapy with the physician’s choice of cabozantinib or vandetanib (LIBRETTO-531 clinicaltrialgov NCT04211337) in patients with *RET*-mutant MTC [18]. Patients treated with selpercatinib presented higher PFS and treatment failure-free survival compared with those treated with cabozantinib or vandetanib (HR 0.28, *p* < 0.001) [18]. Pralsetinib efficacy and safety were established in a phase 1/2 study, an open-label clinical trial (ARROW, clinicaltrialgov NCT03037385 [43]). This treatment induced a disease control in 93% and 100% of MTC patients already treated with vandetanib and cabozantinib, respectively, and 100% in *RET* fusion-positive TC patients [43]. However, Hadoux et al. have recently showed that 16/46 patients treated with highly RET inhibitors (i.e., selpercatinib and pralsetinib) discontinued the treatment for disease progression [44]. This escape phenomenon could be due to a bypass mechanism of resistance (RAS and MYC genes mutations and FGFR2 and ALK fusions) and RET mutations intrinsically resistant to these highly RET inhibitors (solvent front- and hind-region mutations) [44].

Other therapies such as dabrafenib/trametenib and larotrectinib are currently approved by regulatory agency for tumors harboring the *BRAF* mutation and *NTRK* gene fusion, respectively [45]. In TC cases harboring these genetic alterations, as observed for the other treatments, both therapies induced only a partial and transient response [46,47]. Interestingly, alterations of components of the BCL2 pathway—such as the copy number gain of myeloid cell leukemia 1 (MCL1) and loss of cyclin-dependent kinase inhibitor 2A (CDK2NA)—induced resistance to BRAF inhibition, which can be restored in the presence of a BCL2 inhibitor [48]. Moreover, mutations occurring in the PI3K pathway can fulfil resistance to BRAF inhibition [49].

It is worth noting that although targeted therapies obtained significant results in preclinical and clinical studies, a resistance phenomenon still occurs and these canonical preclinical models were able to demonstrate only few resistance mechanisms, since they are not fully able to recapitulate the tumor ecosystem. Cancer research represents one of the main challenges in biomedical research. While some diseases have reliable models, research in other diseases does not. This is the case of some pulmonary diseases, such as cystic fibrosis, in which, despite the availability of many animal models, only pigs and ferrets can reproduce the lung diseases [50]. Furthermore, mice models and even non-human primates often do not fully recapitulate the human diseases, due to species-specific differences in receptors distribution, protease expression, and host immune responses [51,52]. In this scenario, 3D preclinical model can add other significant insights about cancer—and in particular TC—pathogenesis and response to therapies.

## 3. Three-Dimensional Cell Culture Models

**Figure 1:**
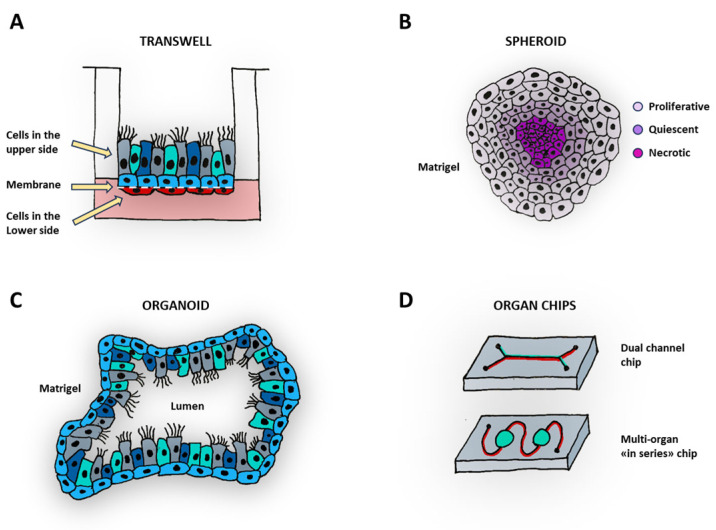
Three-dimensional models used in cancer research. (**A**) Transwell models can be used for co-culturing different cell types or inducing stem cell-to-tissue differentiation, and interface it with other cell types. The cartoon shows a differentiated epithelium cultured under an air–liquid interface condition in the apical side of the porous membrane (dashed line) and another cell type (e.g., endothelial cells) in the lower side of the membrane. Different cell types are color-coded. (**B**) Cancer cells cultured under specific conditions (e.g., Matrigel) can form spheroids and recapitulate the proliferative, quiescent and necrotic regions (related to the oxygen gradient) of solid tumors. (**C**) Epithelial stem cells can form organoids when cultured in Matrigel and under specific conditions. In the organoid culture, the cells can polarize and differentiate in several cell types to recapitulate the structure and architecture of the organ of origin. Different cell types are color coded. (**D**) The organ-on-a-chip technology involves the use of microfluidic device, cultured by dynamic conditions. These devices can be composed by several channels to interface different cell types (**top**) or can host “in series” (**bottom**) cell cultured in different compartments.

Traditional 2D cell cultures have been employed for more than half century to evaluate the impact of drugs on cancer cell survival and death. Nonetheless, these cultures do not allow the study of intricate interactions among cancer cells and the stromal or vascular components, not mentioning immune cells. Three-dimensional culture models, such as co-cultures on transwells, spheroids, and organoids, offer a great advantage over conventional 2D cell cultures (Figure 1).

In the field of cancer research, assays on transwell (Figure 1A) have been employed to explore the migration and invasion of cancer cells through pores of a semipermeable membrane coated with an extracellular matrix (ECM) [53]. Cultures on transwells have the advantage to allow a basolateral feeding, which is important in cell-to-tissue differentiation [54,55]. Moreover, the other side of the porous membrane can host a secondary cell population, like endothelial cells. In cancer research, this model offers the possibility of drug administration from a basolateral compartment and through an endothelium, thus mimicking a systemic delivery. Moreover, transwells with a large pore-size membrane (3–8 µm) have been employed in cancer cell migration and invasion assays [53], as well as immune cell migration [56].

Cancer cell spheroids (Figure 1B) also represent a good model to mimic interactions among tumor cells and the surrounding tissue microenvironment. When allowed to grow to a considerable size, spheroids can establish oxygen and nutrient gradients, leading to the formation of a necrotic core similar to that found in poorly vascularized tumor central regions [57,58,59]. Spheroid cultures have been also exploited in thyroid cancer research to study cellular proliferation, viability, hypoxia, ECM, cytoskeleton and thyroid differentiation in a more reliable setting compared to conventional 2D cultures [60].

The development of organoid culture technology (Figure 1C) provided innovative approach in vitro for drug discovery and personalized medicine [61]. This technique consists of culturing normal or cancerous epithelial stem cells—isolated from patients—in ECM gels under 3D culture conditions through self-organizing organotypic structures. Organoids can recapitulate the structural and functional characteristics of the organs in vivo and allow the interaction of multiple human cell types, offering advantages over canonical 2D cultures and often over animal models. Human intestine organoids were first successfully cultured by Sato et al. [62], mimicking villus-like structures starting from Lgr^5+^ stem cells. Since then, the organoid models in the gut have been improved [63], and several other organs such as lung [64], thyroid [65], stomach [66], heart [67], kidney [68,69], liver [70], brain [71], and retina [72] have been modeled.

Both organoids and spheroids led to considerable improvements in cancer research using a 3D and more reliable context [73] compared to 2D cultures. They are structurally distinct, primarily differing in the cells from whom they originate. Spheroids can reproduce solid, avascular tumors, mimicking the various phenotypes of a tumor mass based on oxygen gradient, thus predicting drug delivery more realistically than 2D cell cultures [74]. However, they are generated from cells with same characteristics (e.g., a cancer cell line), suffering from limitation of the low complexity [73]. Organoids are more complex cultures, often originating from pluripotent stem cells [74] or patient xenografts containing a wider variety of initial cells [75]. This leads to the reproduction of highly complex 3D structures. On the other hand, organoids suffer from significant limitations, in particular size reproducibility; however, new techniques are facing this challenge [73].

## 4. Organoid Cultures Modeling Thyroid Cancer

Few examples of organoid culture in TC have been recently reported. Chen et al. established an organoid culture derived from patients with PTC [76]. They collected and isolated cells from freshly resected PTC (n = 14) and nodular thyroid goiter (NTG) (n = 4). They were able to generate organoids in 10/14 PTC and 3/4 NTG samples and they compared them with parental tumors (primitive tumors). Interestingly, PTC organoids recapitulated histopathological features and the expression of thyroid transcription factor-1, thyroglobulin and proliferation marker (Ki-67) of parental tumors. Moreover, they observed a concordance between genetic mutations observed in organoid cultures and parental tumors, as well as somatic copy number variations. Finally, they used this model to perform a drug sensitivity assay with the most common treatments used in advanced/metastatic TC [76] (Table 1). The same group used this technology to assess the efficacy of BRAF inhibitors (vemurafenib and dabrafenib) alone and in combination with MEK (trametinib and selumetinib), receptor tyrosine kinase inhibitors (sorafenib, lenvatinib, cabozantinib, vandetanib and sunitinib) or chemotherapy (doxorubicin, vincristine, paclitaxel and cisplatin) (Table 1) [76]. They observed that these combinations could be more effective than single treatment [76]. Although from a clinical point of view it is not simple to use this model in all kinds of TC, this model could give several interesting insights in specific cases (e.g., metastatic PTC or MTC at diagnosis) or hystotypes (e.g., ATC).

Lasolle et al. performed an organoid culture model from PTC cells engineered from thyroid cells obtained from mouse embryonic stem cells (mESC) (Table 1) [78]. They generated mESC with a *BRAF* mutation and applied a thyroid differentiation protocol to obtain thyroid cells with the *BRAF* mutation. Using these cells, they built an organoid culture and observed that combination therapy based upon MAPK and PI3K pathway inhibition decreased proliferation, increased apoptosis, impaired ERK phosphorylation and induced the re-expression of thyroid differentiation markers (Na^+^/I^−^ symporter, thyroglobulin, thyroid stimulating hormone receptor and thyroid peroxidase) more than MAPK inhibition alone [78]. This 3D cell culture could model TC harboring major thyroid oncogenes to assess mechanisms associated with its development and progression, thyroid re-differentiation and drug screening, although it does not assess the potential effect of the tumor ecosystem on these phenomena. Moreover, organoids but also spheroids and transwell cultures are static models. This means that they lack mechanical forces, such as fluid shear stress, hydrostatic pressure, and tissue deformation, which can significantly impact the behavior of cancer cells [81]. A static culture also means that cells are not fed continuously by fresh nutrients and through a vascular endothelium like in the human body. In this regard, in the absence of blood flow, organoids cannot receive sufficient nutrients or oxygen to support larger structures or more complex tissue functions. Moreover, they do not allow to study immune cell recruitment, thus the inflammatory response, as well as the physiological dosing of therapeutic agents under investigation. Furthermore, we should also highlight that the other main limitations of the organoid cultures are the high variability in their organization (e.g., uncontrolled size), poor reproductively, and inadequate complexity [82].

## 5. Three-Dimensional Culture Models on the Way: Present and Future Perspectives

Organ-on-chip technology (Figure 1D) has revolutionized preclinical research, providing great advantages over static cultures given by a dynamic flow and the possibility to incorporate shear forces and mechanical strain. Manufactured by numerous companies, these devices consist of channels or compartments typically separated by semipermeable membranes [83]. The use of these microfluidic devices allows the reproduction of the microphysiology of various organs and tissues [84]. These micro-devices, usually made in polycarbonate, polystyrene, or polydimethylsiloxane (PDMS), can host several cell populations in one or more channels, which can communicate with each other through a semipermeable membrane or through an ECM [84,85,86]. The cells can also differentiate into tissues, further enhancing the complexity of the culture models. This innovative technology enables precise control on several parameters, such as fluid shear stress, chemical concentration gradients, pH variations, and temperature alterations. The use of these devices in medical research facilitates the exploration of cell patterning, tissue–tissue interfaces, and organ–organ interactions, mirroring the intricate structures of human and animal bodies. Consequently, it reproduces the complexity of biological microenvironments in vitro associated with high fidelity.

Thanks to all these advantages, organ-on-a-chip technology has gained approval for drug testing as an alternative to animal models (“FDA Modernization ACT 2.0” [87]). This not only reduces costs but also addresses ethical concerns associated with animal testing. Moreover, the ability to recreate a vascular channel and perfuse blood cells, allowing their migration from the vascular channel to an inflamed epithelium, renders this technology well-suited for conducting studies on inflammation [88,89]. This technology has been exploited to faithfully reproduce the function of several tissues and organs, including bronchi [90,91], small airways [92], alveoli [93], the gastrointestinal tract [89,94], the blood–brain barrier [95], the liver [96], the vagina [97] and others. The precision in replicating organs allows for highly translatable data from organ-on-chips to humans. Consequently, organ-on-chip technology has garnered significant attention in the research field, particularly in the study of various diseases such as cystic fibrosis [91], environmental enteric dysfunction [94], viral infections [90,93,98], and cancer [99], where the organ-on-a-chip technology represents one of the best 3D in vitro models. The availability of a channel with a vascular endothelium offers the possibility to study the cell invasion and the metastatic process [100]. At the same time, it represents the most faithful way for drug delivery, thus mimicking in the best way the systemic administration of anticancer drug treatment [100].

During the last years, this technology was successfully applied to different cancers model. In the field of lung cancer, Hassell et al. [100] cultured H1975 human non-small cell lung cancer cells simultaneously with a 100-fold higher number of primary lung epithelial cells on the top channel of a microfluidic chip. The cells were lined by a microvascular endothelium in the bottom channel and let differentiate under an air–liquid condition. The tumor growth, as well as its invasion and response to tyrosine kinase inhibitors, was evaluated using this model [100]. Zhang et al. used a three-channel chip to model breast cancer [101]. The model was composed of human umbilical vein endothelial cells (HUVEC), and MDA-MB-231 breast cancer cells. The authors show that cancer cells can cause angiogenesis, since they observed self-assembled structures of the HUVEC. In this model, they tested the efficacy of the VEGFR inhibitor apatinib. The authors also tested the anti-cancer drugs 5-fluorouracil and doxorubicin, highlighting the minor efficacy in the 3D system when compared to 2D conventional cell cultures [101]. A two-chamber metastasis-on-a-chip was established for investigating intestine cancer migration to the liver. For this model, Skardal et al. tested the ability of the colon carcinoma cell line HCT-116 to invade the liver cells HepG2 [102]. The Epithelial–Mesenchymal transition (EMT) was also studied in a microdevice setting. In this regards, cancer-related fibroblasts were able to induce EMT when co-cultured with colon or pancreatic cancer cells [103,104]. In the field of breast and liver cancer research, Ozkan et al. developed a vascularized breast cancer connected in series with a healthy or tumorigenic liver-on-a-chip. In this study, the co-culture of MDA-MB-231 breast cancer cells, TIME endothelial cells and healthy (THLE-3) or cancer (C3Asub28) liver cells allowed the study of vessel porosity and permeability of the tumor microenvironment [105]. Along these lines, the same research team developed a 3D vascularized hepatocellular carcinoma-on-a-chip, composed of cancer, endothelial, Kupffer and stellate cells. This liver-cancer-on-a-chip model has the aims to study the tumor response to drug treatment and chemotherapy-associated endothelial porosity [106].

## 6. Application of the Organ-on-a-Chip Technology in the Field of Thyroid

Although organ-on-chip technology could produce interesting insights into TC pathogenesis as well as metastasizing phenomenon, currently, there is not any report about its use in TC field. On the other hand, few studies have investigated thyroid function using this technology. In particular, Carvalho et al. developed a thyroid organoid-on-a-chip to evaluate the effect of endocrine disruptors on thyroid hormone synthesis (Table 1) [79]. First, they obtained thyroid organoids starting from mESC; thereafter, they evaluated hormone synthesis in static and dynamic conditions inside an organ-on-a-chip device. They observed that, although thyroid cells have a lower expression of thyroperoxidase and thyroid-stimulating-hormone receptor in dynamic condition compared with static one, thyroxine production was much higher in dynamic condition [79]. The exposure of these organoids to 10 × 10^−6^ M mbenzo[k]fluoranthene (BKF) for 24 h induced a dramatic change in gene expression, resulting in significant decrease in thyroperoxidase and Na^+^/I^−^ symporter expression. Interestingly, the same exposure of these cells in 2D condition induced a less dramatic change in gene expression, emphasizing that thyroid organ-on-a-chip technology could be a very sensitive in vitro assay to evaluate endocrine disruptors effect on thyroid follicle activity.

Karwelat et al. described the use of organ-on-a-chip technology to assess the effect of many compounds on the thyroid–liver axis, building a thyroid follicular-like architecture model, associated with liver 3D spheroids (Table 1) [80]. They observed that methimazole and 6-propylthiouracil inhibits thyroxine synthesis by thyroid follicles but not its liver conjugation with glucuronide; on the other hand, inducers of liver biotransformation increased thyroxine glucuronidation, without impairing its synthesis by thyroid follicles [80]. Intriguingly, this model could be able to assess direct effects of compound on thyroid function ruling out indirect ones by liver and vice versa. Of note, these two examples showed that this technology could be useful in thyroid field to assess the effect of many compounds on its function as well as its interactions with other organs.

## 7. Conclusions

The emergence of systemic therapies has shown promise in managing advanced/metastatic TC, although resistance remains a significant challenge. Traditional preclinical models, such as of traditional 2D cell cultures and animal ones, have limitations in capturing the complexities of TC and its response to therapies. For this reason, 3D models, such as transwell, spheroids, organoids, and organ-on-chip technology could better represent the tumor complexities, providing insights into TC pathogenesis and response to treatments. Among them, organ-on-chip technology is a revolutionary approach in preclinical research, allowing for dynamic, physiologically relevant conditions that mimic the microphysiology of organs. While this technology has been successfully applied in various fields, including cancer research, its application in the TC field is yet to be totally explored. For this reason, this review could be considered a call for further use of this technology also in TC research, given its powerful and unprecedent reliability in reproducing organ pathophysiology and predicting drug efficacy.

## Figures and Tables

**Table 1:** Preclinical 3D culture models and their application in thyroid cancer.

Publications	3D Model	Thyroid Cells Origin	Aim	Modeled Drugs
Chen D et al. [76]	Organoid culture	Patient-derived	Anti-neoplastic drug sensitivity assay	Sorafenib, lenvatinib, cabozantinib, vandetanib, sunitinib, axitinib, pazopanib, motesanib, apatinib, imatinib, vemurafenib, dabrafenib, gefitinib, everolimus, temsirolimus, AZD8055, selumetinib, trametinib, doxorubicin, cisplatin, bleomycin, vincristine, paclitaxel
Chen D et al. [77]				Vemurafenib and dabrafenib, as single agents and combined with selumetinib, trametinib, sorafenib, lenvatinib, cabozantinib, vandetanib, sunitinib, doxorubicin, vincristine, paclitaxel cisplatin
Lasolle H et al. [78]		mESC-derived		Dabrafenib, trametinib and MEK inhibitor as single agents and combined with PI3K inhibitor
Carvalho DJ et al. [79]	Organ-on-chip		Endocrine disruptor effects	benzo[k]fluoranthene
Karwelat D et al. [80]		Rat-derived	Liver–thyroid axis interactions	Methimazole, 6-propylthiouracil, rifampicin, pregnenolone 16α-carbonitrile, fipronil sulfone, β-naphthoflavone, phenobarbital and clofibrate
